# Perceived Neighborhood Trust, Health Behaviors, and Metabolic Syndrome Among Middle-Aged and Older Chinese Adults: A Cross-Sectional Study

**DOI:** 10.3390/bs16071151

**Published:** 2026-07-09

**Authors:** Yifan Chen, Yongqi Wang, Tingting Yang, Xilin He, Xianbin Ding, Yaoyue Hu

**Affiliations:** 1 School of Public Health, Chongqing Medical University, Chongqing 400016, China; 2024111656@stu.cqmu.edu.cn (Y.C.); 2023121707@stu.cqmu.edu.cn (Y.W.); 2023111595@stu.cqmu.edu.cn (T.Y.); 2024121726@stu.cqmu.edu.cn (X.H.); 2 Chongqing Municipal Center for Disease Control and Prevention, Chongqing 400707, China

**Keywords:** perceived neighborhood trust, metabolic syndrome, health behavior, mediation

## Abstract

Metabolic syndrome (MetS) is rising rapidly in China. Perceived neighborhood trust may be associated with MetS through health behaviors, but evidence is limited. Using baseline data from 15,512 adults aged ≥45 years from the Chongqing sub-cohort of China Multi-Ethnic Cohort, we examined the association between perceived neighborhood trust and MetS and explored potential indirect associations through smoking, alcohol consumption, and leisure-time physical activity. Compared with low perceived neighborhood trust, medium trust (OR = 0.78, 95% CI: 0.63–0.96) and high trust (OR = 0.79, 95% CI: 0.65–0.96) were associated with lower odds of MetS, whereas very high trust was not. Exploratory mediation analyses showed a small inverse indirect association for smoking status (high vs. low trust: OR = 0.988, 95% CI: 0.974–0.998) and for smoking pack-years (medium vs. low: OR = 0.989, 95% CI: 0.976–0.999; high vs. low: OR = 0.985, 95% CI: 0.971–0.995; very high vs. low: OR = 0.985, 95% CI: 0.970–0.996) in the total sample. Subgroup analyses suggested similar small inverse indirect associations for smoking among men and adults aged 45–59 years, while alcohol consumption and leisure-time physical activity showed no indirect associations. These findings highlight the potential relevance of neighborhood trust to metabolic health and suggest that future prevention strategies in China may need to consider broader social and contextual factors beyond individual health behaviors.

## 1. Introduction

Metabolic syndrome (MetS) is a cluster of interrelated metabolic abnormalities, including abdominal obesity, hyperglycemia, dyslipidemia, and elevated blood pressure (Alberti et al., 2009). Globally, MetS prevalence has risen alarmingly alongside economic development and lifestyle shifts (Aggarwal et al., 2024), with particularly steep increases observed in low- and middle-income countries (H. H. Wang et al., 2020). In China, MetS prevalence nearly doubled between 2002 and 2012 (9.5% to 18.7%) (He et al., 2019), and more recent data from the China Nutrition and Health Surveillance (2015–2017) reported a standardized prevalence of 31.1% (F. Yao et al., 2021). Given MetS’s well-established role as a major risk factor for cardiovascular disease (CVD) and type 2 diabetes mellitus (T2DM) (Kassi et al., 2011), its prevention and management have become a critical public health priority in China, especially at the community level, where lifestyle interventions can be effectively implemented.

Social trust—a core component of social capital—refers to reasonable, necessary, and beneficial expectations individuals hold regarding others’ behaviors in social interactions, typically expressed as confidence (Schwerter & Zimmermann, 2020). As a social resource, social trust may promote social integration, cooperation, and societal harmony (Delhey & Newton, 2003). Prior studies have linked higher social trust to better physical and mental health outcomes, greater life satisfaction, and longer longevity (Delhey & Newton, 2003; Min, 2020). Individuals with higher trust may be more likely to utilize social networks within the community to mitigate the health impacts of adverse life events (e.g., unemployment) (Harpham et al., 2002), and engage in group activities to optimize health resource allocation and facilitate information exchange (e.g., acquisition of nutrition knowledge), thereby enhancing health outcomes (Campos-Matos et al., 2016). Crucially, social trust not only boosts social participation and social activities by expanding social networks (Kiani et al., 2023), but also alleviates chronic stress by reducing anxiety and fear about others’ behaviors (Abbott & Freeth, 2008). In the present study, we focus on perceived neighborhood trust, assessed by whether residents believe that people living in their neighborhood can be trusted. This measure captures a neighborhood-based and self-perceived dimension of social trust, reflecting trust in the immediate social environment where daily interactions, social norms, and health-related behaviors are shaped (Diez Roux & Mair, 2010).

Evidence linking perceived neighborhood trust with MetS is limited. A Korean cohort study found that higher social trust was associated with lower incidence of MetS (Park et al., 2020). Using data from the Health and Retirement Study (HRS), a neighborhood cohesion measure that included neighborhood trust was associated with lower cardiometabolic risk both cross-sectionally and longitudinally (Robinette et al., 2018). Another cross-sectional study from the U.S. using the same neighborhood cohesion measure reported that higher neighborhood cohesion was associated with lower MetS severity in women but not in men (Moniruzzaman et al., 2025). Emerging evidence also suggest that social trust is associated with individual MetS components, including lower rates of obesity (Wu et al., 2018), hypertension (Kiani et al., 2023), hyperglycemia (T. Xue et al., 2021), and dyslipidemia (Yang et al., 2025). However, the association between perceived neighborhood trust and MetS may not be linear. While neighborhood trust may facilitate social support, perceived safety, and health-related information exchange (Kawachi et al., 1997), it is also possible that high-trust environments may reinforce local norms that are not necessarily health-promoting, including smoking in social settings, alcohol-centered gatherings, and shared meals that may encourage overconsumption of energy-dense foods (Christakis, 2007). The association may also reflect threshold or diminishing-return patterns, in which moderate levels of trust are sufficient to provide metabolic benefits. Health behaviors may help explain the observed association between perceived neighborhood trust and MetS. Social trust may foster health-promoting behaviors that prevent or delay MetS onset (Jahangiry et al., 2015), and improve social participation that enhances health awareness, access to healthcare, and adherence to health behaviors and treatment (Palafox et al., 2017). Prior evidence indicates that social trust is correlated with reduced smoking and alcohol consumption, increased physical activity, better sleep quality, and healthier weight maintenance (X. Xue & Cheng, 2017). Among these behaviors, smoking, alcohol consumption, and physical activity are particularly relevant because they are socially patterned and closely related to metabolic health (Cockerham, 2005). Smoking is associated with insulin resistance, central adiposity, dyslipidemia, and systemic inflammation (Messner & Bernhard, 2014), and remains highly prevalent among Chinese men (Chan et al., 2023). Alcohol consumption may contribute to abdominal obesity, hypertension, dyslipidemia, and impaired glucose metabolism (Freiberg et al., 2004). Physical activity is generally associated with lower MetS risk through improved insulin sensitivity, weight control, and lipid metabolism (Pedersen & Saltin, 2015). Thus, these behaviors are plausible behavioral links through which perceived neighborhood trust may be associated with MetS.

China provides an important setting for this investigation. Although China generally exhibits high social trust (Steinhardt, 2012), it has undergone rapid urbanization and social transformation (Xinhua, 2024), eroding traditional trust structures based on acquaintance networks amid growing interactions with strangers (Xu et al., 2022). Concurrently, the rapid rise in MetS prevalence in China is directly linked to economic growth, urbanization, and consequent lifestyle and dietary shifts (Li et al., 2016), These contextual shifts make it important to examine how perceived neighborhood trust is associated with MetS in the Chinese population and whether health behaviors are involved in this association. In addition, smoking (Chen et al., 2015), drinking (Millwood et al., 2017), and physical activity (Du et al., 2014) are strongly patterned by gender roles, social expectations, health status, and life stage in China. Gender and age may therefore modify these associations. Because smoking (M. Wang et al., 2019), drinking (Im et al., 2023), and social participation are socially patterned in China (R. Wang et al., 2019), the extent to which neighborhood trust is translated into health-related behaviors may differ across men and women. Moreover, middle-aged adults may have more active work- and community-based social networks and may be more responsive to social norms around smoking and drinking (Im et al., 2023), whereas older adults may have more comorbidities, different patterns of social participation, and more health-related behavior changes (R. Wang et al., 2019).

Therefore, using baseline cross-sectional data from the Chongqing sub-cohort of the China Multi-Ethnic Cohort (CMEC) Study, we examined the association between perceived neighborhood trust and MetS among adults aged 45 years and older. We further explored whether perceived neighborhood trust linking MetS through smoking, alcohol consumption, and leisure-time physical activity and whether it varied by sex and age.

## 2. Methods

### 2.1. Study Population

We used the baseline data (i.e., cross-sectional) from the Chongqing sub-cohort of the China Multi-Ethnic Cohort Study. Between September 2018 and January 2019, 23,329 Han Chinese adults aged 30–79 years were recruited from 13 districts/counties in Chongqing via a multi-stage, stratified cluster sampling method. The baseline survey collected data on sociodemographic characteristics, lifestyle, chronic diseases, mental health, and physical examination (Zhao et al., 2021). The CMEC study was approved by the Ethics Committee of Sichuan University (No. K2016038), and the Chongqing sub-cohort was further approved by the Ethics Committee of Chongqing Municipal Center for Disease Control and Prevention (No. 2017-001). All participants provided written informed consent. Further details on the study design can be found elsewhere (Zhao et al., 2021). Given that MetS predominantly emerges in midlife, we included participants aged ≥45 years and excluded those with missing data on study variables (*N* = 15,512, see Figure S1).

### 2.2. Perceived Neighborhood Trust

Perceived neighborhood trust was assessed using a single-item question: “Do you agree that most people who live in the neighborhood can be trusted?”. Responses were captured on a five-point Likert scale: strongly agree, agree, averagely agree, disagree, strongly disagree. This item was adapted from a set of questionnaires used to measure social cohesion and trust (Sampson et al., 1997), and has been used to explore the relationship between social trust and health (Ziersch et al., 2005; Feng et al., 2016). Because the numbers of participants reporting “disagree” and especially “strongly disagree” were small (disagree: *N* = 627, 4.04%; strongly disagree: *N* = 46, 0.30%), these two categories were combined. Perceived neighborhood trust levels were categorized as very high (strongly agree), high (agree), medium (average) and low (disagree/strongly disagree).

### 2.3. Metabolic Syndrome

MetS was identified using self- reports, anthropometric measurements, and blood test results based on the Guideline for the Prevention and Treatment of Diabetes Mellitus in China (Chinese Diabetes Society, 2025). At least three of the following criteria were required for diagnosis: (1) abdominal obesity: waist circumference ≥ 90 cm in men and ≥85 cm in women; (2) hyperglycemia: self-reported doctor diagnosis of diabetes or fasting blood glucose ≥ 6.1 mmol/L (Chinese Diabetes Society, 2021); (3) elevated blood pressure: self-reported diagnosis of hypertension or systolic blood pressure ≥ 130 mmHg/diastolic blood pressure ≥ 85 mmHg (J. G. Wang, 2025); (4) fasting triglyceride (TG) ≥ 1.70 mmol/L; and (5) fasting high density lipoprotein cholesterol (HDL-C) < 1.04 mmol/L.

### 2.4. Health Behaviors

Health behaviors included smoking, alcohol drinking, and leisure-time physical activity. Smoking status was categorized as never, ever, and current smoking. Cumulative smoking exposure was quantified using pack-years. For participants who reported using loose tobacco rather than cigarettes, we converted grams of tobacco into pack equivalents using 12.5 g of loose tobacco per pack-equivalent (Wood et al., 2005). Frequency of drinking any type of alcoholic beverage in the past year was categorized into never, occasionally, <1/week, 1–2/week, 3–5/week, and daily. Frequency of leisure-time physical activity in the past year was grouped as never, 1–3/month, 1–2/week, 3–5/week, and daily.

### 2.5. Covariates

Covariates included socio-demographic characteristics, anxiety, and depression. Socio-demographic characteristics covered age, gender, marital status (married/cohabiting vs. unmarried/divorced/separated/widowed), educational attainment (≤primary school, lower secondary school, ≥upper secondary school), occupation (manual vs. non-manual), and annual pre-tax household income (<¥12,000, ¥12,000–59,999, ¥60,000–99,999, and ≥¥100,000). Symptoms of anxiety and depression were assessed using the 2-item Generalized Anxiety Disorder Scale (GAD-2) (Plummer et al., 2016) and the Patient Health Questionnaire 2 (PHQ-2) (Kroenke et al., 2003), respectively. A total score ≥ 3 indicated probable anxiety or depression (Hughes et al., 2018, Villarreal-Zegarra et al., 2023). Body Mass Index (BMI) was not included as a covariate due to its high correlation with abdominal obesity (a component of MetS) and potential for overadjustment (Zhou et al., 2023).

### 2.6. Statistical Analysis

The association between perceived neighborhood trust and MetS was analyzed using logistic regression. Perceived neighborhood trust was entered as a categorical variable, with low trust as the reference group. Model 1 adjusted for sociodemographic characteristics, anxiety, and depression, and Model 2 further adjusted for health behaviors. Following the traditional difference method of mediation analysis (VanderWeele, 2016), we compared the estimated association between perceived neighborhood trust and MetS before and after adjustment for health behaviors. Attenuation of the association after adjustment for health behaviors was considered suggestive of a potential indirect association. We further conducted exploratory simple mediation and multiple parallel mediation analyses. We reported the total, direct and indirect effects for each trust contrast (medium vs. low, high vs. low, and very high vs. low), along with their 95% confidence intervals (CI) using the bias-corrected percentile bootstrap method (5000 replications). An effect was considered statistically significant if its 95% CI did not include zero. The proportion mediated was not reported because it can be mathematically unstable when the total association is close to, non-significant, or in the opposite direction to the indirect association, potentially yielding negative or extremely large values. Given substantial differences in health behaviors by sex and age (Deeks et al., 2009). we performed sensitivity analyses stratified by gender and age group. All data analyses were conducted using R version 4.4.3 (R Core Team, 2024) with the “lavaan” package version 0.6-19 for mediation analyses.

## 3. Results

Of the 15,512 participants, 12,790 (82.45%) did not have MetS, while 2722 (17.45%) met the diagnostic criteria for MetS (Table 1). The prevalence was higher in men (21.08%) than in women (14.26%). The majority reported high or very high perceived neighborhood trust (74.89%), with only 4.34% reporting low trust. Smoking was reported by 28.57% of participants, and among smokers, 7.15% had a cumulative exposure of ≥40 pack-years. Approximately half of participants reported no alcohol consumption (48.37%) or no leisure-time physical activity (42.01%). Marked gender differences were observed for smoking and alcohol drinking, but not for physical activity. Most participants were married or cohabiting, had education attainment below upper secondary school, worked in non-manual occupations, and reported a pre-tax household income < ¥60,000. Probable anxiety or depression was uncommon.

Compared with low perceived neighborhood trust, medium trust (OR: 0.78, 95% CI: 0.63–0.96) and high trust (OR: 0.79, 95% CI: 0.65–0.96) were associated with a lower risk of MetS after adjustment for sociodemographic factors, anxiety, and depression (Model 1, Table 2). However, very high perceived neighborhood trust was not associated with MetS (OR: 0.82, 95% CI: 0.66–1.02). These associations remained basically unchanged after further adjustment for health behaviors (Model 2), suggesting that smoking, alcohol consumption, and leisure-time physical activity did not substantially explain the association between perceived neighborhood trust and MetS when using traditional difference method of mediation analysis. Stratified analyses showed that this inverse association was mainly observed among participants aged 45–59 years, but not within either gender subgroup or among participants aged ≥60 years (see Tables S1 and S2 for details).

In the exploratory mediation analyses, the total effects were consistent with the logistic regression results that medium and high perceived neighborhood trust were associated with lower odds of MetS, whereas very high perceived neighborhood trust was not. In the simple mediation models, the indirect effects through health behaviors were generally very small (Table 3). For smoking status, compared with low perceived neighborhood trust, an indirect effect was observed only for high perceived neighborhood trust in the total sample (OR: 0.988, 95% CI: 0.974–0.998) and among men (OR: 0.985, 95% CI: 0.968–0.996). When smoking pack-years was used as an alternative smoking measure, indirect effects were observed for medium (OR: 0.989, 95% CI: 0.976–0.999), high (OR: 0.985, 95% CI: 0.971–0.995), and very high perceived neighborhood trust (OR: 0.985, 95% CI: 0.970–0.996) in the total sample. Among men, the corresponding indirect effects were 0.984 (95% CI: 0.968–0.996), 0.980 (95% CI: 0.962–0.991), and 0.980 (95% CI: 0.961–0.994), respectively. Among participants aged 45–59 years, an indirect effect through smoking pack-years was observed only for high perceived neighborhood trust (OR: 0.979, 95% CI: 0.957–0.995). No significant indirect effects through alcohol drinking or physical activity were observed.

In exploratory multiple parallel mediation models, the indirect effect through smoking status remained only for high perceived neighborhood trust in the total sample (Table 4, OR: 0.988, 95% CI: 0.977–0.998) and among men (OR: 0.985, 95% CI: 0.968–0.996). When using smoking pack-years, indirect effects were observed in the total sample for high trust (Table 5, OR: 0.985, 95% CI: 0.973–0.997) and very high perceived neighborhood trust (OR: 0.985, 95% CI: 0.972–0.998). Among men, indirect effects through smoking pack-years were also observed for medium (OR: 0.984, 95% CI: 0.970–0.998), high (OR: 0.980, 95% CI: 0.965–0.994), and very high trust (OR: 0.980, 95% CI: 0.964–0.996). Among participants aged 45–59 years, the indirect effect through smoking pack-years was observed only for high perceived neighborhood trust (OR: 0.979, 95% CI: 0.960–0.998). Similarly, no indirect effects through alcohol drinking or physical activity were observed in the total sample or any subgroup.

## 4. Discussion

In this large cross-sectional study of 15,512 middle-aged and older Chinese adults, moderate-to-high levels of perceived neighborhood trust were associated with lower odds of MetS, while no clear association was observed at the highest level of trust. Exploratory mediation analyses suggested that the selected health behaviors explained only a limited proportion of the observed association. Small inverse indirect associations were observed mainly for smoking exposures, whereas no indirect associations were found for alcohol drinking or leisure-time physical activity.

Our finding of an inverse association between perceived neighborhood trust and MetS is broadly consistent with previous studies. A large longitudinal cohort study from Korean reported that adults in the highest quintile of social trust had a 12% lower risk of incident MetS compared to those in the lowest quintile (Park et al., 2020). In the Health and Retirement Study, higher neighborhood cohesion, which included neighborhood trust, was associated with lower cardiometabolic risk both cross-sectionally and four years later (Robinette et al., 2018). In addition, although a cross-sectional study from the U.S. using the same neighborhood cohesion measure found an inverse association between neighborhood cohesion and MetS severity in women but not in men (Moniruzzaman et al., 2025), our gender-stratified analysis did not show a significant association in either gender. Several explanations may account for the observed inverse association between perceived neighborhood trust and MetS. Higher trust may buffer chronic psychological stress by increasing perceived safety, reciprocity, and social support, thereby reducing sustained hypothalamic–pituitary–adrenal axis activation and cortisol dysregulation (Stewart-Knox, 2005). Moreover, high-trust environments may facilitate the formation and maintenance of health-related social norms, making it easier for individuals to adopt and sustain healthier behaviors. However, the absence of an association at the highest level of trust should be interpreted cautiously and may indicate a non-linear pattern, particularly among adults aged 45–59 years. One possible hypothesis is a threshold or diminishing-returns pattern, whereby moderate level of neighborhood trust may be sufficient to provide perceived safety, informal support, and opportunities for health-related information exchange (Villalonga-Olives & Kawachi, 2017), and additional increases in trust may provide limited additional metabolic benefit. Another possible hypothesis is that very high trust may reinforce local social norms that are not necessarily health-promoting (Cislaghi & Heise, 2018), such as smoking in social settings (Christakis & Fowler, 2008), alcohol-centered gatherings (Rosenquist et al., 2010), or shared meals that encourage overconsumption of energy-dense foods (Herman, 2015). These explanations nevertheless remain speculative and are best regarded as hypotheses to be examined in longitudinal studies.

In our exploratory mediation analyses, the behavior-related indirect associations were small and were mainly limited to smoking-related measures. For smoking status, an inverse indirect association was observed only for high versus low perceived neighborhood trust in the total sample and among men. When smoking pack-years was used as an alternative measure, inverse indirect associations were more consistently observed. However, the magnitude of these associations was very small, with ORs close to 1.00, and their statistical significance should not be interpreted as indicating meaningful practical or biological relevance. The small indirect associations should therefore not be interpreted as evidence that smoking is beneficial. Rather, they suggest, at most, a weak exploratory statistical indirect association in which higher perceived neighborhood trust was associated with lower smoking exposure, and lower smoking exposure was associated with lower odds of MetS. Smoking is a well-established risk factor for metabolic abnormalities (Messner & Bernhard, 2014) and remains highly prevalent among Chinese men. (Chan et al., 2023) The somewhat more consistent smoking-related indirect associations among men may reflect greater variation in smoking exposure and greater statistical power to detect smoking-related associations in this subgroup. In addition, higher perceived neighborhood trust may be associated with lower smoking exposure through stronger health-promoting social norms, informal social monitoring, or greater social reinforcement for smoking reduction or cessation (Lindström, 2003; Lindström, 2010; Bottorff et al., 2018). This possibility may be particularly relevant for men, as smoking behavior in men is often more closely embedded in social interactions and may be more responsive to perceived social status and belonging. By contrast, we did not observe a similar indirect association in women. This difference may partly reflect gender differences in smoking prevalence, smoking-related social contexts, and the limited variation in smoking exposure among women in this sample. Given the small effect sizes and the cross-sectional design, these interpretations should be regarded as tentative and hypothesis-generating.

The smoking-related indirect associations also appeared to vary by age, with the most consistent indirect associations observed among adults aged 45–59 years, particularly when smoking pack-years was used. The difference between smoking status and smoking pack-years suggests that cumulative smoking exposure may be captured more adequately by pack-years than by current smoking status, although both measures may not fully capture smoking intensity, duration, or changes over time. This may reflect a life stage at which social networks remain active and influential, while awareness of chronic disease risk becomes more salient (Lahiri et al., 2025). In this context, social trust may more readily translate into encouragement to reduce smoking or comply with cessation advice (Kasiviswanathan et al., 2025). By contrast, the absence of a clear smoking-related indirect association among older adults may indicate that long term cumulative smoking damage, comorbidities, and age-related metabolic decline reduce the extent to which behavioral changes are reflected in current metabolic status (S. Yao et al., 2024). However, because multiple subgroup analyses and mediation models were conducted, the possibility of chance findings and inflated type I error cannot be ruled out. Therefore, these subgroup-specific findings should be interpreted cautiously and considered hypothesis-generating rather than confirmatory.

By contrast, we did not observe indirect associations involving alcohol drinking or leisure-time physical activity. These findings should be interpreted cautiously because both behaviors were measured relatively crudely. Alcohol drinking was assessed by frequency only and did not capture quantity, binge drinking, beverage type, drinking context, or drinking pattern (Im et al., 2023). Similarly, leisure-time physical activity was measured only by self-reported frequency over the past year and did not include duration, intensity, total volume, or occupational, household, and transportation-related activity (Bull et al., 2020) Therefore, the behavioral measures used in this study may not adequately capture the aspects of alcohol use and physical activity most relevant to metabolic health (Pattyn et al., 2013). In addition, self-reported alcohol drinking and physical activity may be affected by reporting bias (Kilian et al., 2020; Strain et al., 2020), and broad behavioral categories may have diluted heterogeneous associations across different patterns of drinking and physical activity (Bull et al., 2020, Im et al., 2023). In addition, reverse causation is possible in this cross-sectional setting as individuals with metabolic abnormalities may reduce alcohol consumption or increase physical activity after diagnosis or medical advice. Therefore, the absence of indirect associations through alcohol drinking and physical activity should not be interpreted as evidence that these behaviors are irrelevant to the relationship between perceived neighborhood trust and MetS.

More broadly, the small indirect associations and their restriction mainly to smoking exposures suggest that smoking, alcohol drinking, and leisure-time physical activity captured only a limited part of the processes potentially linking perceived neighborhood trust with MetS. Perceived neighborhood trust may be embedded in a broader set of social, behavioral, psychosocial, and environmental factors (Ehsan et al., 2019). These factors may include diet, sleep, chronic stress, social support, health literacy, healthcare access, medication use, neighborhood environments, and local social norms (Hill-Briggs et al., 2020). Thus, the selected health behaviors should be viewed as exploratory indicators rather than a comprehensive representation of the underlying processes. Importantly, this limitation is not simply a matter of omitting additional mediators. Rather, the very small and inconsistent indirect associations may reflect the difficulty of representing a complex social and metabolic process using a restricted set of observable behavioral variables. As a result, our mediation framework may provide an oversimplified representation of the underlying pathways linking perceived neighborhood trust and MetS. The small magnitude of the indirect associations also highlights the importance of distinguishing statistical significance from practical or clinical relevance, particularly in a large sample. This interpretation is consistent with recent arguments that important dimensions of complexity may remain hidden when analytic frameworks rely primarily on a restricted set of standardized or readily measurable indicators (Cesare et al., 2026).

Our study has several strengths. To our knowledge, it is the first study that investigated the association between perceived neighborhood trust and MetS and to explore indirect associations through multiple health behaviors in a large sample of middle-aged and older Chinese adults. The large sample size allowed us to examine heterogeneity by sex and age. In addition, by considering smoking, alcohol consumption, and leisure-time physical activity simultaneously, our study provides a more comprehensive exploratory assessment of behavior-related indirect associations than analyses focusing on a single behavior. However, several limitations should be noted. First, the cross-sectional design precludes conclusions about temporality or causality. Perceived neighborhood trust, health behaviors, and MetS were measured at the same time point, and the temporal ordering required for causal mediation could not be established. Reverse causation is plausible, as individuals with MetS or related health concerns may change their health behaviors or social engagement after diagnosis, medical advice, or awareness of health risks. Therefore, the mediation results should be interpreted as exploratory statistical indirect associations rather than evidence of causal mechanisms. Second, perceived neighborhood trust was measured using a single item. Although this item has been used in previous studies (Ziersch et al., 2005; Feng et al., 2016), it captures only one neighborhood-based aspect of trust and cannot distinguish generalized interpersonal trust, institutional trust, trust in close social ties, or other dimensions of social trust (Schilke et al., 2021). Third, health behaviors were self-reported and measured crudely. These measurement limitations may have limited our ability to detect indirect associations through alcohol drinking or leisure-time physical activity. Finally, residual confounding is possible because important individual-level and environmental factors were not included or fully measured, such as diet, sleep, medication use, family history of cardiometabolic disease, healthcare access, neighborhood environment, and other contextual exposures. These factors may influence perceived neighborhood trust, health behaviors, and MetS simultaneously and may bias the observed associations. Future longitudinal studies using more comprehensive and validated measures of perceived neighborhood trust, health behaviors, diet, clinical information, and environmental exposures are needed to clarify temporality and potential mechanisms.

In conclusion, moderate-to-high perceived neighborhood trust was associated with lower odds of MetS in middle-aged and older Chinese adults, while no clear association was observed at the highest level of trust. Exploratory mediation analyses suggested that the selected health behaviors explained only a limited proportion of this association, with small indirect associations limited to smoking. These findings highlight the potential relevance of neighborhood trust to metabolic health and suggest that future prevention strategies in China may need to consider broader social and contextual factors beyond individual health behaviors.

## Figures and Tables

**Table 1:** Sample characteristics.

	Total (*N* = 15,512)	Men (*N* = 7476)	Women (*N* = 8036)
Perceived neighbourhood trust (*N*, %)			
Low	673 (4.34)	319 (4.27)	354 (4.41)
Medium	3222 (20.77)	1488 (19.90)	1734 (21.58)
High	9662 (62.29)	4753 (63.58)	4909 (61.09)
Very high	1955 (12.60)	916 (12.25)	1039 (12.93)
Metabolic syndrome (*N*, %)			
No	12,790 (82.45)	5900 (78.92)	6890 (85.74)
Yes	2722 (17.55)	1576 (21.08)	1146 (14.26)
Abdominal obesity ^a^ (*N*, %)			
No	9898 (63.81)	4840 (64.74)	5058 (62.94)
Yes	5614 (36.19)	2636 (35.26)	2978 (37.06)
Elevated blood pressure ^d^ (*N*, %)			
No	10,276 (66.25)	4621 (61.81)	5655 (70.37)
Yes	5236 (33.75)	2855 (38.19)	2381 (29.63)
Elevated glucose ^e^ (*N*, %)			
No	12,249 (78.96)	5772 (77.21)	6477 (80.60)
Yes	3263 (21.04)	1704 (22.79)	1559 (19.40)
Hypertriglyceridemia ^g^ (*N*, %)			
<1.70	10,635 (68.56)	4876 (65.22)	5759 (71.67)
≥1.70	4877 (31.44)	2600 (34.78)	2277 (28.33)
High-density lipoprotein ^f^ (*N*, %)			
≥1.04	14,147 (91.20)	6455 (86.34)	7692 (95.72)
<1.04	1365 (8.80)	1021 (13.66)	344 (4.28)
Age (*N*, %)			
45–49	4565 (29.43)	2036 (27.23)	2529 (31.47)
50–54	2911 (18.77)	1374 (18.38)	1537 (19.13)
55–59	2078 (13.40)	1009 (13.50)	1069 (13.30)
60–64	2328 (15.01)	1190 (15.92)	1138 (14.16)
≥65	3630 (23.40)	1867 (24.97)	1763 (21.94)
Marital status (*N*, %)			
Married/cohabiting	13,505 (87.06)	6823 (91.27)	6682 (83.15)
Unmarried/divorced/separated/widowed	2007 (12.94)	653 (8.73)	1354 (16.85)
Education (*N*, %)			
≤Primary school	6476 (41.75)	2694 (36.04)	3782 (47.06)
Lower secondary school	5040 (32.49)	2558 (34.22)	2482 (30.89)
≥Upper secondary school	3996 (25.76)	2224 (29.75)	1772 (22.05)
Occupation (*N*, %)			
Manual	3635 (23.43)	2241 (29.98)	1394 (17.35)
Non-manual	11,877 (76.57)	5235 (70.02)	6642 (82.65)
Household income (*N*, %)			
<12,000	2139 (13.79)	1049 (14.03)	1090 (13.56)
12,000–59,999	7728 (49.82)	3564 (47.67)	4164 (51.82)
60,000–99,999	3164 (20.40)	1555 (20.80)	1609 (20.02)
≥100,000	2481 (15.99)	1308 (17.50)	1173 (14.60)
Health behaviors			
Smoking status (*N*, %)			
Never	11,081 (71.44)	3118 (41.71)	7963 (99.09)
Ever	1191 (7.68)	1175 (15.72)	16 (0.20)
Current	3240 (20.89)	3183 (42.58)	57 (0.71)
Smoking pack-years ^h^ (*N*, %)			
0	11,081 (71.53)	3118 (41.82)	7963 (99.09)
<10	744 (4.80)	700 (9.39)	44 (0.55)
10–20	804 (5.19)	790 (10.60)	14 (0.17)
20–30	901 (5.82)	890 (11.94)	11 (0.14)
30–40	852 (5.50)	849 (11.39)	3 (0.04)
≥40	1109 (7.16)	1108 (14.86)	1 (0.01)
Alcohol drinking (*N*, %)			
Never	7503 (48.37)	2314 (30.95)	5189 (64.57)
Occasionally	5079 (32.74)	2642 (35.34)	2437 (30.33)
<1/week	484 (3.12)	341 (4.56)	143 (1.78)
1–2/week	592 (3.82)	488 (6.53)	104 (1.29)
3–5/week	605 (3.90)	547 (7.32)	58 (0.72)
daily	1249 (8.05)	1144 (15.30)	105 (1.31)
Physical activity (*N*, %)			
Never	6672 (43.01)	3398 (45.45)	3274 (40.74)
1–3/month	719 (4.64)	383 (5.12)	336 (4.18)
1–2/week	1295 (8.35)	634 (8.48)	661 (8.23)
3–5/week	1096 (7.07)	517 (6.92)	579 (7.21)
Daily	5730 (36.94)	2544 (34.03)	3186 (39.65)
Anxiety ^b^ (*N*, %)			
No	14,765 (95.18)	7211 (96.46)	7554 (94.00)
Yes	747 (4.82)	265 (3.54)	482 (6.00)
Depression ^c^ (*N*, %)			
No	14,844 (95.69)	7201 (96.32)	7643 (95.11)
Yes	668 (4.31)	275 (3.68)	393 (4.89)

^a^ Abdominal obesity: waist circumference ≥ 90 cm (men) or ≥85 cm (women). ^b^ Anxiety: GAD-2 score ≥ 3. ^c^ Depression: PHQ-2 score ≥ 3. ^d^ Elevated blood pressure: self-reported hypertension or SBP ≥ 130 mmHg or DBP ≥ 85 mmHg. ^e^ Elevated glucose: self-reported diabetes or FPG ≥ 6.1 mmol/L. ^f^ Low HDL-C: HDL-C < 1.04 mmol/L. ^g^ Hypertriglyceridemia: TG ≥ 1.70 mmol/L. ^h^ Smoking pack-years: calculated using 12.5 g loose tobacco per pack-equivalent.

**Table 2:** Associations between perceived neighborhood trust and metabolic syndrome (*N* = 15,512).

	Model 1 OR (95% CI)	Model 2 OR (95% CI)
Perceived neighbourhood trust		
Low	Ref	Ref
Medium	0.78 * (0.63–0.96)	0.78 * (0.63–0.96)
High	0.79 * (0.65–0.96)	0.79 * (0.65–0.96)
Very high	0.82 (0.66–1.02)	0.82 (0.66–1.02)
Smoking status		
Never		Ref
Ever		1.26 * (1.07–1.48)
Current		1.31 * (1.15–1.48)
Alcohol drinking		
Never		Ref
Occasionally		0.88 * (0.79–0.97)
<1/week		1.17 (0.93–1.48)
1–2/week		1.35 * (1.10–1.65)
3–5/week		1.34 * (1.09–1.64)
daily		0.81 * (0.68–0.95)
Physical activity		
Never		Ref
1–3/month		1.14 (0.92–1.40)
1–2/week		1.06 (0.90–1.25)
3–5/week		1.13 (0.95–1.34)
Daily		1.08 (0.98–1.19)

OR: odds ratio; CI, confidence interval; Ref: reference category. Model 1: Adjusted for age, gender, marital status, education, occupation, household income, anxiety and depression. Model 2: Model 1+health behaviors. * 95% confidence interval does not include 1.00 (*p* < 0.05).

**Table 3:** Simple mediation analysis of health behaviors on the association between perceived neighborhood trust and metabolic syndrome.

	Total (*N* = 15,512)	Men (*N* = 7476)	Women *(N* = 8036)	Age 45–59 Years (*N* = 9554)	Age ≥ 60 Years (*N* = 5958)
Perceived neighborhood trust → Smoking status → MetS					
Indirect effect					
Medium vs. Low	0.997 (0.985–1.008)	0.995 (0.979–1.007)	1.006 (0.978–1.918)	1.005 (0.986–1.028)	0.997 (0.976–1.005)
High vs. Low	0.988 * (0.974–0.998)	0.985 * (0.968–0.996)	1.003 (0.972–1.714)	0.986 (0.965–1.004)	0.996 (0.975–1.005)
Very high vs. Low	0.991 (0.975–1.001)	0.988 (0.971–1.001)	0.997 (0.902–1.058)	0.993 (0.970–1.015)	0.996 (0.973–1.005)
Direct effect					
Medium vs. Low	0.872 * (0.771–0.980)	0.890 (0.748–1.033)	0.882 (0.703–1.065)	0.840 * (0.710–0.983)	0.946 (0.792–1.138)
High vs. Low	0.885 * (0.783–0.978)	0.880 (0.748–1.033)	0.898 (0.709–1.071)	0.851 * (0.731–0.990)	0.948 (0.798–1.124)
Very high vs. Low	0.903 (0.787–1.017)	0.900 (0.756–1.079)	0.908 (0.720–1.114)	0.950 (0.800–1.120)	0.857 (0.704–1.038)
Total effect					
Medium vs. Low	0.870 * (0.771–0.980)	0.886 (0.747–1.044)	0.888 (0.749–1.057)	0.844 * (0.724–0.990)	0.943 (0.791–1.130)
High vs. Low	0.875 * (0.783–0.978)	0.866 (0.739–1.018)	0.901 (0.774–1.059)	0.840 * (0.719–0.978)	0.944 (0.794–1.117)
Very high vs. Low	0.895 (0.787–1.017)	0.889 (0.745–1.061)	0.905 (0.763–1.088)	0.943 (0.793–1.114)	0.853 (0.702–1.033)
Perceived neighborhood trust → Smoking pack-years → MetS					
Indirect effect					
Medium vs. Low	0.989 * (0.976–0.999)	0.984 * (0.968–0.996)	1.005 (0.980–1.886)	0.988 (0.967–1.005)	0.991 (0.967–1.001)
High vs. Low	0.985 * (0.971–0.995)	0.980 ** (0.962–0.991)	1.003 (0.976–1.678)	0.979 * (0.957–0.995)	0.991 (0.966–1.001)
Very high vs. Low	0.985 * (0.970–0.996)	0.980 * (0.961–0.994)	0.999 (0.927–1.067)	0.986 (0.963–1.006)	0.988 (0.961–1.001)
Direct effect					
Medium vs. Low	0.879 * (0.771–0.980)	0.900 (0.756–1.079)	0.883 (0.704–1.065)	0.854 (0.724–0.998)	0.952 (0.797–1.143)
High vs. Low	0.888 * (0.783–0.978)	0.884 (0.755–1.039)	0.899 (0.715–1.068)	0.858 * (0.739–0.999)	0.953 (0.802–1.132)
Very high vs. Low	0.908 (0.787–1.017)	0.908 (0.760–1.089)	0.906 (0.738–1.112)	0.956 (0.807–1.131)	0.864 (0.708–1.050)
Total effect					
Medium vs. Low	0.870 * (0.771–0.980)	0.886 (0.747–1.045)	0.888 (0.747–1.058)	0.844 * (0.724–0.990)	0.943 (0.791–1.130)
High vs. Low	0.875 * (0.783–0.978)	0.866 (0.739–1.018)	0.901 (0.775–1.059)	0.840 * (0.719–0.978)	0.944 (0.794–1.117)
Very high vs. Low	0.895 (0.787–1.017)	0.889 (0.745–1.061)	0.905 (0.763–1.089)	0.943 (0.793–1.114)	0.853 (0.702–1.033)
Perceived neighborhood trust → Alcohol drinking ^a^ → MetS					
Indirect effect					
Medium vs. Low	0.999 (0.994–1.001)	1.005 (0.999–1.018)	0.997 (0.984–1.007)	1.000 (0.992–1.004)	0.999 (0.989–1.004)
High vs. Low	1.001 (0.999–1.006)	0.999 (0.989–1.005)	1.007 (0.999–1.022)	1.000 (0.998–1.005)	1.003 (0.999–1.016)
Very high vs. Low	1.001 (0.998–1.007)	0.997 (0.985–1.002)	1.009 (0.999–1.025)	1.000 (0.997–1.006)	1.006 (0.998–1.022)
Direct effect					
Medium vs. Low	0.870 * (0.771–0.980)	0.881 (0.742–1.042)	0.890 (0.749–1.058)	0.844 * (0.722–0.991)	0.944 (0.792–1.133)
High vs. Low	0.875 * (0.783–0.978)	0.867 (0.741–1.018)	0.895 (0.767–1.052)	0.840 * (0.720–0.977)	0.941 (0.790–1.116)
Very high vs. Low	0.894 (0.787–1.017)	0.892 (0.746–1.064)	0.897 (0.758–1.077)	0.943 (0.791–1.111)	0.848 (0.699–1.028)
Total effect					
Medium vs. Low	0.870 * (0.771–0.980)	0.886 (0.747–1.045)	0.888 (0.747–1.058)	0.844 * (0.724–0.990)	0.943 (0.791–1.130)
High vs. Low	0.875 * (0.783–0.978)	0.866 (0.739–1.018)	0.901 (0.775–1.059)	0.840 * (0.719–0.978)	0.944 (0.794–1.117)
Very high vs. Low	0.895 (0.787–1.017)	0.889 (0.745–1.061)	0.905 (0.763–1.089)	0.943 (0.793–1.114)	0.853 (0.702–1.033)
Perceived neighborhood trust → Physical activity → MetS					
Indirect effect					
Medium vs. Low	1.003 (0.999–1.010)	1.008 (0.997–1.019)	0.999 (0.989–1.006)	1.003 (0.997–1.013)	1.007 (0.996–1.018)
High vs. Low	1.005 (0.998–1.013)	1.011 (0.999–1.022)	0.998 (0.985–1.009)	1.005 (0.994–1.017)	1.009 (0.997–1.021)
Very high vs. Low	1.004 (0.998–1.013)	1.011 (0.999–1.023)	0.998 (0.985–1.010)	1.005 (0.994–1.017)	1.010 (0.997–1.023)
Direct effect					
Medium vs. Low	0.867 * (0.771–0.980)	0.879 (0.742–1.038)	0.889 (0.748–1.057)	0.841 * (0.718–0.987)	0.936 (0.785–1.121)
High vs. Low	0.871 * (0.783–0.978)	0.857 (0.733–1.003)	0.903 (0.775–1.064)	0.836 * (0.719–0.972)	0.936 (0.787–1.108)
Very high vs. Low	0.891 (0.787–1.017)	0.880 (0.740–1.050)	0.907 (0.766–1.092)	0.938 (0.794–1.110)	0.844 (0.698–1.024)
Total effect					
Medium vs. Low	0.870 * (0.771–0.980)	0.886 (0.747–1.045)	0.888 (0.747–1.058)	0.844 * (0.724–0.990)	0.943 (0.791–1.130)
High vs. Low	0.875 * (0.783–0.978)	0.866 (0.739–1.018)	0.901 (0.774–1.059)	0.840 * (0.719–0.978)	0.944 (0.794–1.117)
Very high vs. Low	0.895 (0.787–1.017)	0.889 (0.745–1.061)	0.905 (0.763–1.088)	0.943 (0.793–1.114)	0.853 (0.702–1.033)

Values are presented as ORs with 95% confidence intervals; MetS: metabolic syndrome. Mediation analyses used bias-corrected percentile bootstrap with 5000 replications. Models adjusted for age, gender, marital status, education, occupation, and household income, probable anxiety, and probable depression. * 95% confidence interval does not include 1.00 (*p* < 0.05). ** 95% confidence interval does not include 1.00 (*p* < 0.01). ^a^ Due to small number of some alcohol drinking frequency groups, alcohol drinking frequency was dichotomized.

**Table 4:** Multiple parallel mediation analysis of smoking status, alcohol drinking, and physical activity on the association between perceived neighborhood trust and metabolic syndrome.

	Total (*N* = 15,512)	Men (*N* = 7476)	Women (*N* = 8036)	Age 45–59 Years (*N* = 9554)	Age ≥ 60 Years (*N* = 5958)
Indirect effect					
Smoking status					
Medium vs. Low	0.997 (0.986–1.008)	0.995 (0.979–1.007)	1.006 (0.986–1.028)	1.005 (0.986–1.028)	0.997 (0.976–1.005)
High vs. Low	0.988 * (0.977–0.998)	0.985 * (0.968–0.996)	1.003 (0.972–1.714)	0.986 (0.965–1.004)	0.996 (0.975–1.005)
Very high vs. Low	0.991 (0.978–1.004)	0.988 (0.970–1.001)	0.997 (0.902–1.058)	0.993 (0.970–1.015)	0.996 (0.973–1.005)
Alcohol drinking					
Medium vs. Low	1.000 (0.998–1.001)	0.999 (0.993–1.004)	0.999 (0.989–1.006)	1.001 (0.999–1.007)	1.001 (0.998–1.011)
High vs. Low	1.001 (0.998–1.003)	0.997 (0.988–1.001)	1.006 (0.998–1.019)	0.998 (0.992–1.001)	1.004 (0.999–1.016)
Very high vs. Low	1.001 (0.997–1.004)	0.996 (0.985–1.000)	1.005 (0.998–1.018)	0.999 (0.992–1.001)	1.005 (0.999–1.019)
Physical activity					
Medium vs. Low	1.003 (0.998–1.008)	1.008 (0.997–1.019)	0.999 (0.989–1.006)	1.003 (0.997–1.013)	1.007 (0.999–1.022)
High vs. Low	1.005 (0.998–1.012)	1.011 (0.999–1.022)	0.998 (0.985–1.009)	1.005 (0.994–1.017)	1.009 (0.999–1.024)
Very high vs. Low	1.004 (0.998–1.011)	1.011 (0.999–1.023)	0.998 (0.985–1.010)	1.005 (0.994–1.017)	1.010 (0.999–1.027)
Direct effect					
Medium vs. Low	0.869 * (0.771–0.981)	0.884 (0.746–1.046)	0.885 (0.685–1.145)	0.837 * (0.708–0.979)	0.938 (0.787–1.126)
High vs. Low	0.881 * (0.787–0.985)	0.873 (0.747–1.022)	0.895 (0.695–1.154)	0.848 * (0.709–0.988)	0.936 (0.789–1.108)
Very high vs. Low	0.899 (0.785–1.023)	0.894 (0.751–1.072)	0.905 (0.693–1.179)	0.947 (0.799–1.118)	0.844 (0.694–1.025)
Total effect					
Medium vs. Low	0.870 * (0.771–0.980)	0.886 (0.747–1.045)	0.888 (0.749–1.057)	0.844 * (0.723–0.990)	0.943 (0.793–1.114)
High vs. Low	0.875 * (0.783–0.978)	0.866 (0.742–1.018)	0.901 (0.776–1.058)	0.840 * (0.722–0.978)	0.944 (0.793–1.118)
Very high vs. Low	0.895 (0.787–1.017)	0.889 (0.744–1.062)	0.905 (0.767–1.097)	0.943 (0.793–1.114)	0.853 (0.693–1.033)

Values in parentheses are95% confidence intervals. Multiple parallel mediation models included smoking status, alcohol drinking, and physical activity simultaneously. Models adjusted for age, gender, marital status, education, occupation, household income, probable anxiety, and probable depression. Bootstrap with 5000 replications. * 95% confidence interval does not include 1.00 (*p* < 0.05).

**Table 5:** Multiple parallel mediation analysis of smoking pack-years, alcohol drinking, and physical activity on the association between perceived neighborhood trust and metabolic syndrome.

	Total (*N* = 15,512)	Men (*N* = 7476)	Women (*N* = 8036)	Age 45–59 Years (*N* = 9554)	Age ≥ 60 Years (*N* = 5958)
Indirect effect					
Smoking pack-years					
Medium vs. Low	0.989 (0.970–1.001)	0.984 * (0.970–0.998)	1.005 (0.828–1.220)	0.988 (0.970–1.007)	0.991 (0.975–1.007)
High vs. Low	0.985 * (0.973–0.997)	0.980 ** (0.965–0.994)	1.005 (0.829–1.218)	0.979 * (0.960–0.998)	0.991 (0.975–1.007)
Very high vs. Low	0.985 * (0.972–0.998)	0.980 * (0.964–0.996)	0.999 (0.826–1.209)	0.986 (0.966–1.007)	0.988 (0.969–1.007)
Alcohol drinking					
Medium vs. Low	0.999 (0.998–1.001)	0.999 (0.994–1.005)	0.999 (0.990–1.007)	1.001 (0.997–1.004)	1.001 (0.995–1.007)
High vs. Low	1.001 (0.998–1.003)	0.997 (0.991–1.003)	1.006 (0.997–1.015)	0.998 (0.994–1.003)	1.004 (0.996–1.011)
Very high vs. Low	1.001 (0.997–1.004)	0.996 (0.989–1.003)	1.005 (0.995–1.015)	0.999 (0.994–1.003)	1.005 (0.996–1.014)
Physical activity					
Medium vs. Low	1.003 (0.998–1.008)	1.008 (0.997–1.019)	0.999 (0.991–1.006)	1.003 (0.995–1.011)	1.007 (0.996–1.018)
High vs. Low	1.005 (0.996–1.012)	1.011 (0.999–1.022)	0.998 (0.986–1.010)	1.005 (0.994–1.016)	1.009 (0.997–1.024)
Very high vs. Low	1.004 (0.998–1.011)	1.011 (0.999–1.023)	0.998 (0.986–1.010)	1.005 (0.993–1.016)	1.010 (0.997–1.027)
Direct effect					
Medium vs. Low	0.876 * (0.776–0.989)	0.893 (0.754–1.058)	0.886 (0.685–1.145)	0.851 * (0.724–0.999)	0.944 (0.787–1.132)
High vs. Low	0.884 * (0.790–0.989)	0.878 (0.751–1.026)	0.896 (0.695–1.154)	0.855 * (0.733–0.998)	0.941 (0.792–1.118)
Very high vs. Low	0.904 (0.794–1.028)	0.902 (0.757–1.080)	0.904 (0.693–1.179)	0.953 (0.805–1.129)	0.851 (0.700–1.034)
Total effect					
Medium vs. Low	0.869 * (0.771–0.980)	0.886 (0.747–1.049)	0.888 (0.750–1.051)	0.844 * (0.720–0.989)	0.943 (0.787–1.130)
High vs. Low	0.875 * (0.783–0.977)	0.866 (0.742–1.012)	0.901 (0.769–1.056)	0.840 * (0.722–0.977)	0.944 (0.796–1.120)
Very high vs. Low	0.895 (0.787–1.017)	0.889 (0.746–1.060)	0.905 (0.756–1.084)	0.943 (0.797–1.115)	0.853 (0.703–1.035)

Values in parentheses are95% confidence intervals. Multiple parallel mediation models included smoking status, alcohol drinking, and physical activity simultaneously. Models adjusted for age, gender, marital status, education, occupation, household income, probable anxiety, and probable depression. Bootstrap with 5000 replications. * 95% confidence interval does not include 1.00 (*p* < 0.05). ** *p* < 0.01.

## Data Availability

Restrictions apply to the availability of these data. Data were obtained from the China Multi-Ethnic Cohort (CMEC) Study and are available upon reasonable request to the corresponding authors of the CMEC Study, subject to approval by the CMEC data access committee. The authors of this manuscript do not have permission to share the data directly. The present study analyzed data collected from the Chongqing Cohort of the CMEC Study. No new data were collected directly from participants for this paper, and no additional participant recruitment was conducted.

## Supplementary Materials

The following supporting information can be downloaded at: https://www.mdpi.com/article/10.3390/bs16071151/s1, Figure S1: Analytical sample flowchart. Table S1: Age-stratified association results. Table S2: Gender-stratified association results.

## References

[B1-behavsci-16-01151] Abbott S., Freeth D. (2008). Social capital and health: Starting to make sense of the role of generalized trust and reciprocity. Journal Health Psychol.

[B2-behavsci-16-01151] Aggarwal R., Ostrominski J. W., Vaduganathan M. (2024). Prevalence of cardiovascular-kidney-metabolic syndrome stages in US adults, 2011–2020. JAMA.

[B3-behavsci-16-01151] Alberti K. G., Eckel R. H., Grundy S. M., Zimmet P. Z., Cleeman J. I., Donato K. A., Fruchart J. C., James W. P., Loria C. M., Smith S. C. (2009). Harmonizing the metabolic syndrome: A joint interim statement. Circulation.

[B4-behavsci-16-01151] Bottorff J. L., Oliffe J. L., Sarbit G., Sharp P., Kelly M. T. (2018). Smoke-free men: Competing and connecting to quit. American Journal of Health Promotion.

[B5-behavsci-16-01151] Bull F. C., Al-Ansari S. S., Biddle S., Borodulin K., Buman M. P., Cardon G., Carty C., Chaput J.-P., Chastin S., Chou R., Dempsey P. C., DiPietro L., Ekelund U., Firth J., Friedenreich C. M., Garcia L., Gichu M., Jago R., Katzmarzyk P. T., Willumsen J. F. (2020). World Health Organization 2020 guidelines on physical activity and sedentary behaviour. British Journal of Sports Medicine.

[B6-behavsci-16-01151] Campos-Matos I., Subramanian S. V., Kawachi I. (2016). The ‘dark side’ of social capital: Trust and self-rated health in European countries. European Journal of Public Health.

[B7-behavsci-16-01151] Cesare M., Gray R., Cocchieri A. (2026). Stop silencing nursing complexity: Why standardized nursing terminologies must be heard. Nursing Reports.

[B8-behavsci-16-01151] Chan K. H., Xiao D., Zhou M., Peto R., Chen Z. (2023). Tobacco control in China. The Lancet Public Health.

[B9-behavsci-16-01151] Chen Z., Peto R., Zhou M., Iona A., Smith M., Yang L., Guo Y., Chen Y., Bian Z., Lancaster G., Sherliker P., Pang S., Wang H., Su H., Wu M., Wu X., Chen J., Collins R., Li L. (2015). Contrasting male and female trends in tobacco-attributed mortality in China: Evidence from successive nationwide prospective cohort studies. The Lancet.

[B53-behavsci-16-01151] Chinese Diabetes Society (2021). Guideline for the prevention and treatment of type 2 diabetes mellitus in China (2020 edition). Chinese Journal of Diabetes Mellitus.

[B54-behavsci-16-01151] Chinese Diabetes Society (2025). Guideline for the prevention and treatment of diabetes mellitus in China (2024 edition). Chinese Journal of Diabetes Mellitus.

[B10-behavsci-16-01151] Christakis N. (2007). The spread of obesity in a large social network over 32 years. New England Journal of Medicine.

[B11-behavsci-16-01151] Christakis N., Fowler J. (2008). The collective dynamics of smoking in a large social network. The New England Journal of Medicine.

[B12-behavsci-16-01151] Cislaghi B., Heise L. (2018). Theory and practice of social norms interventions: Eight common pitfalls. Globalization and Health.

[B13-behavsci-16-01151] Cockerham W. C. (2005). Health lifestyle theory and the convergence of agency and structure. Journal of Health and Social Behavior.

[B14-behavsci-16-01151] Deeks A., Lombard C., Michelmore J., Teede H. (2009). The effects of gender and age on health related behaviors. BMC Public Health.

[B15-behavsci-16-01151] Delhey J., Newton K. (2003). Who trusts?: The origins of social trust in seven societies. European Societies.

[B16-behavsci-16-01151] Diez Roux A. V., Mair C. (2010). Neighborhoods and health. Annals of the New York Academy of Sciences.

[B17-behavsci-16-01151] Du H., Li L., Whitlock G., Bennett D., Guo Y., Bian Z., Chen J., Sherliker P., Huang Y., Zhang N., Zheng X., Li Z., Hu R., Collins R., Peto R., Chen Z. (2014). Patterns and socio-demographic correlates of domain-specific physical activities and their associations with adiposity in the China Kadoorie Biobank study. BMC Public Health.

[B18-behavsci-16-01151] Ehsan A., Klaas H. S., Bastianen A., Spini D. (2019). Social capital and health: A systematic review of systematic reviews. SSM—Population Health.

[B19-behavsci-16-01151] Feng Z., Vlachantoni A., Liu X., Jones K. (2016). Social trust, interpersonal trust and self-rated health in China: A multi-level study. International Journal for Equity in Health.

[B20-behavsci-16-01151] Freiberg M. S., Cabral H. J., Heeren T. C., Vasan R. S., Ellison R. C. (2004). Alcohol consumption and the prevalence of the metabolic syndrome in the U.S.: A cross-sectional analysis of data from the third national health and nutrition examination survey. Diabetes Care.

[B21-behavsci-16-01151] Harpham T., Grant E., Thomas E. (2002). Measuring social capital within health surveys: Key issues. Health Policy Plan.

[B22-behavsci-16-01151] He Y., Li Y., Bai G., Zhang J., Fang Y., Zhao L., Zhao W., Yang X., Ding G. (2019). Prevalence of metabolic syndrome and individual metabolic abnormalities in China, 2002–2012. Asia Pacific Journal of Clinical Nutrition.

[B23-behavsci-16-01151] Herman C. P. (2015). The social facilitation of eating. A review. Appetite.

[B24-behavsci-16-01151] Hill-Briggs F., Adler N. E., Berkowitz S. A., Chin M. H., Gary-Webb T. L., Navas-Acien A., Thornton P. L., Haire-Joshu D. (2020). Social determinants of health and diabetes: A scientific review. Diabetes Care.

[B25-behavsci-16-01151] Hughes A. J., Dunn K. M., Chaffee T., Bhattarai J. J., Beier M. (2018). Diagnostic and clinical utility of the GAD-2 for screening anxiety symptoms in individuals with multiple sclerosis. Archives of Physical Medicine and Rehabilitation.

[B26-behavsci-16-01151] Im P. K., Wright N., Yang L., Chan K. H., Chen Y., Guo Y., Du H., Yang X., Avery D., Wang S., Yu C., Lv J., Clarke R., Chen J., Collins R., Walters R. G., Peto R., Li L., Chen Z., Millwood I. Y. (2023). Alcohol consumption and risks of more than 200 diseases in Chinese men. Nature Medicine.

[B27-behavsci-16-01151] Jahangiry L., Shojaeizadeh D., Farhangi M. A., Yaseri M., Mohammad K., Najafi M., Montazeri A. (2015). Interactive web-based lifestyle intervention and metabolic syndrome: Findings from the Red Ruby (a randomized controlled trial). Trials.

[B28-behavsci-16-01151] Kasiviswanathan K., Enticott J., Madawala S., Selamoglu M., Sturgiss E., Barton C. (2025). The impact of smoking status on trust in general practitioners: A nationwide survey of smokers and ex-smokers. Journal Public Health.

[B29-behavsci-16-01151] Kassi E., Pervanidou P., Kaltsas G., Chrousos G. (2011). Metabolic syndrome: Definitions and controversies. BMC Medicine.

[B30-behavsci-16-01151] Kawachi I., Kennedy B. P., Lochner K., Prothrow-Stith D. (1997). Social capital, income inequality, and mortality. American Journal of Public Health.

[B31-behavsci-16-01151] Kiani M. M., Takian A., Farzadfar F., Rezaei S., Zandian H. (2023). The relationships between social capital, metabolic, and behavioral risk factors of non-communicable diseases: A systematic review. Iran Journal Public Health.

[B32-behavsci-16-01151] Kilian C., Manthey J., Probst C., Brunborg G. S., Bye E. K., Ekholm O., Kraus L., Moskalewicz J., Sieroslawski J., Rehm J. (2020). Why is per capita consumption underestimated in alcohol surveys? Results from 39 surveys in 23 European countries. Alcohol and Alcoholism.

[B33-behavsci-16-01151] Kroenke K., Spitzer R. L., Williams J. B. (2003). The patient health questionnaire-2: Validity of a two-item depression screener. Medical Care.

[B34-behavsci-16-01151] Lahiri S., Bingenheimer J. B., Evans W. D., Wang Y., Cislaghi B., Dubey P., Snowden B. (2025). Understanding the mechanisms of change in social norms around tobacco use: A systematic review and meta-analysis of interventions. Addiction.

[B35-behavsci-16-01151] Li R., Li W., Lun Z., Zhang H., Sun Z., Kanu J. S., Qiu S., Cheng Y., Liu Y. (2016). Prevalence of metabolic syndrome in Mainland China: A meta-analysis of published studies. BMC Public Health.

[B36-behavsci-16-01151] Lindström M. (2003). Social capital and the miniaturization of community among daily and intermittent smokers: A population-based study. Preventive Medicine.

[B37-behavsci-16-01151] Lindström M. (2010). Social capital, economic conditions, marital status and daily smoking: A population-based study. Public Health.

[B38-behavsci-16-01151] Messner B., Bernhard D. (2014). Smoking and cardiovascular disease mechanisms of endothelial dysfunction and early atherogenesis. Arteriosclerosis, Thrombosis, and Vascular Biology.

[B39-behavsci-16-01151] Millwood I. Y., Li L., Smith M., Guo Y., Yang L., Bian Z., Lewington S., Whitlock G., Sherliker P., Collins R., Chen J., Peto R., Wang H., Xu J., He J., Yu M., Liu H. (2017). Alcohol consumption in 0.5 million people from 10 diverse regions of China: Prevalence, patterns and socio-demographic and health-related correlates. International Journal of Epidemiology.

[B40-behavsci-16-01151] Min J. (2020). Does social trust slow down or speed up the transmission of COVID-19?. PLoS ONE.

[B41-behavsci-16-01151] Moniruzzaman M., Reid L. A., Jones K. K., Zenk S. N., Vega G. L., Grundy S. M., Sims M., Powell-Wiley T. M., Tamura K. (2025). Multilevel mediators on the associations of neighborhood social environmental factors and severity of metabolic syndrome: The jackson heart study. Journal of the American Heart Association.

[B42-behavsci-16-01151] Palafox B., Goryakin Y., Stuckler D., Suhrcke M., Balabanova D., Alhabib K. F., Avezum A., Bahonar A., Bai X., Chifamba J., Dans A. L., Diaz R., Gupta R., Iqbal R., Ismail N., Kaur M., Keskinler M. V., Khatib R., Kruger A., McKee M. (2017). Does greater individual social capital improve the management of hypertension? Cross-national analysis of 61 229 individuals in 21 countries. BMJ Global Health.

[B43-behavsci-16-01151] Park H., Choi S., Kim K. H., Kang E., Ko A., Park S. M. (2020). Association between social trust and metabolic syndrome in a previously healthy population—A longitudinal cohort study in South Korea. International Journal of Environmental Research and Public Health.

[B44-behavsci-16-01151] Pattyn N., Cornelissen V. A., Eshghi S. R., Vanhees L. (2013). The effect of exercise on the cardiovascular risk factors constituting the metabolic syndrome: A meta-analysis of controlled trials. Sports Medicine.

[B45-behavsci-16-01151] Pedersen B. K., Saltin B. (2015). Exercise as medicine—Evidence for prescribing exercise as therapy in 26 different chronic diseases. Scandinavian Journal of Medicine & Science in Sports.

[B46-behavsci-16-01151] Plummer F., Manea L., Trepel D., McMillan D. (2016). Screening for anxiety disorders with the GAD-7 and GAD-2: A systematic review and diagnostic metaanalysis. General Hospital Psychiatry.

[B47-behavsci-16-01151] R Core Team (2024). R: A language and environment for statistical computing.

[B48-behavsci-16-01151] Robinette J. W., Charles S. T., Gruenewald T. L. (2018). Neighborhood cohesion, neighborhood disorder, and cardiometabolic risk. Social Science & Medicine.

[B49-behavsci-16-01151] Rosenquist J. N., Murabito J., Fowler J. H., Christakis N. A. (2010). The spread of alcohol consumption behavior in a large social network. Annals of Internal Medicine.

[B50-behavsci-16-01151] Sampson R. J., Raudenbush S. W., Earls F. (1997). Neighborhoods and violent crime: A multilevel study of collective efficacy. Science.

[B51-behavsci-16-01151] Schilke O., Reimann M., Cook K. S. (2021). Trust in social relations. Annual Review of Sociology.

[B52-behavsci-16-01151] Schwerter F., Zimmermann F. (2020). Determinants of trust: The role of personal experiences. Games and Economic Behavior.

[B55-behavsci-16-01151] Steinhardt H. (2012). How is high trust in china possible? Comparing the origins of generalized trust in three Chinese societies. Political Studies.

[B56-behavsci-16-01151] Stewart-Knox B. J. (2005). Psychological underpinnings of metabolic syndrome. Proceedings of the Nutrition Society.

[B57-behavsci-16-01151] Strain T., Wijndaele K., Dempsey P. C., Sharp S. J., Pearce M., Jeon J., Lindsay T., Wareham N., Brage S. (2020). Wearable-device-measured physical activity and future health risk. Nature Medicine.

[B58-behavsci-16-01151] VanderWeele T. J. (2016). Mediation analysis: A practitioner’s guide. Annual Review of Public Health.

[B59-behavsci-16-01151] Villalonga-Olives E., Kawachi I. (2017). The dark side of social capital: A systematic review of the negative health effects of social capital. Social Science & Medicine.

[B60-behavsci-16-01151] Villarreal-Zegarra D., Barrera-Begazo J., Otazú-Alfaro S., Mayo-Puchoc N., Bazo-Alvarez J. C., Huarcaya-Victoria J. (2023). Sensitivity and specificity of the Patient Health Questionnaire (PHQ-9, PHQ-8, PHQ-2) and General Anxiety Disorder scale (GAD-7, GAD-2) for depression and anxiety diagnosis: A cross-sectional study in a Peruvian hospital population. BMJ Open.

[B61-behavsci-16-01151] Wang H. H., Lee D. K., Liu M., Portincasa P., Wang D. Q. (2020). Novel insights into the pathogenesis and management of the metabolic syndrome. Pediatric Gastroenterology, Hepatology & Nutrition.

[B62-behavsci-16-01151] Wang J. G. (2025). Chinese guidelines for the prevention and treatment of hypertension (2024 revision). Journal of Geriatric Cardiology.

[B63-behavsci-16-01151] Wang M., Luo X., Xu S., Liu W., Ding F., Zhang X., Wang L., Liu J., Hu J., Wang W. (2019). Trends in smoking prevalence and implication for chronic diseases in China: Serial national cross-sectional surveys from 2003 to 2013. The Lancet Respiratory Medicine.

[B64-behavsci-16-01151] Wang R., Chen Z., Zhou Y., Shen L., Zhang Z., Wu X. (2019). Melancholy or mahjong? Diversity, frequency, type, and rural-urban divide of social participation and depression in middle- and old-aged Chinese: A fixed-effects analysis. Social Science & Medicine.

[B65-behavsci-16-01151] Wood D. M., Mould M. G., Ong S. B., Baker E. H. (2005). Pack year smoking histories: What about patients who use loose tobacco?. Tobacco Control.

[B66-behavsci-16-01151] Wu Y. H., Moore S., Dube L. (2018). Social capital and obesity among adults: Longitudinal findings from the Montreal neighborhood networks and healthy aging panel. Preventive Medicine.

[B67-behavsci-16-01151] Xinhua (2024). China sees rising urbanization rate over past 75 years. China Daily.

[B68-behavsci-16-01151] Xu G., Liao Y., Jiang Y., Xu P., Yang L., Huang W., Zhang M., Wu R. (2022). The impacts of urban environments on community trust of the low-income group: A case study for the pearl river delta region. Land.

[B69-behavsci-16-01151] Xue T., Rahmaty Z., McConnell E., Xu Y., Corazzini K. (2021). Impacts of social capital factors on blood glucose control and depressive symptoms. Innov Aging.

[B70-behavsci-16-01151] Xue X., Cheng M. (2017). Social capital and health in China: Exploring the mediating role of lifestyle. BMC Public Health.

[B71-behavsci-16-01151] Yang T., Zhu X., Wang Y., Tang Y., Tang X., Xue B., Ding X., Hu Y. (2025). Cross-sectional relationship between social trust and health in middle-aged and older Chinese adults: Can social trust explain the education-health link?. Geriatrics & Gerontology International.

[B72-behavsci-16-01151] Yao F., Bo Y., Zhao L., Li Y., Ju L., Fang H., Piao W., Yu D., Lao X. (2021). Prevalence and influencing factors of metabolic syndrome among adults in China from 2015 to 2017. Nutrients.

[B73-behavsci-16-01151] Yao S., Colangelo L. A., Perry A. S., Marron M. M., Yaffe K., Sedaghat S., Lima J. A. C., Tian Q., Clish C. B., Newman A. B., Shah R. V., Murthy V. L. (2024). Implications of metabolism on multi-systems healthy aging across the lifespan. Aging Cell.

[B74-behavsci-16-01151] Zhao X., Hong F., Yin J., Tang W., Zhang G., Liang X., Li J., Cui C., Li X. (2021). Cohort profile: The China Multi-Ethnic Cohort (CMEC) study. International Journal of Epidemiology.

[B75-behavsci-16-01151] Zhou J., He R., Shen Z., Zhang Y., Gao X., Dejiquzong, Xiao X., Zhang T., Yang D., Wang Y., Song H., Guo Y., Li S., Chen G., Yin J., Zhao X. (2023). Altitude and metabolic syndrome in China: Beneficial effects of healthy diet and physical activity. Journal of Global Health.

[B76-behavsci-16-01151] Ziersch A. M., Baum F. E., Macdougall C., Putland C. (2005). Neighbourhood life and social capital: The implications for health. Social Science & Medicine.

