# Supplementary material for: Perceived Neighborhood Trust, Health Behaviors, and Metabolic Syndrome Among Middle-Aged and Older Chinese Adults: A Cross-Sectional Study
Source: Behav Sci (Basel). 2026 Jul 9;16(7):1151. doi: 10.3390/bs16071151 (PMC13405394; doi:10.3390/bs16071151)
Supplement: Supplementary file 1 [file behavsci-16-01151-s001.zip › behavsci-4371771-supplementary.pdf]

Supplementary Materials

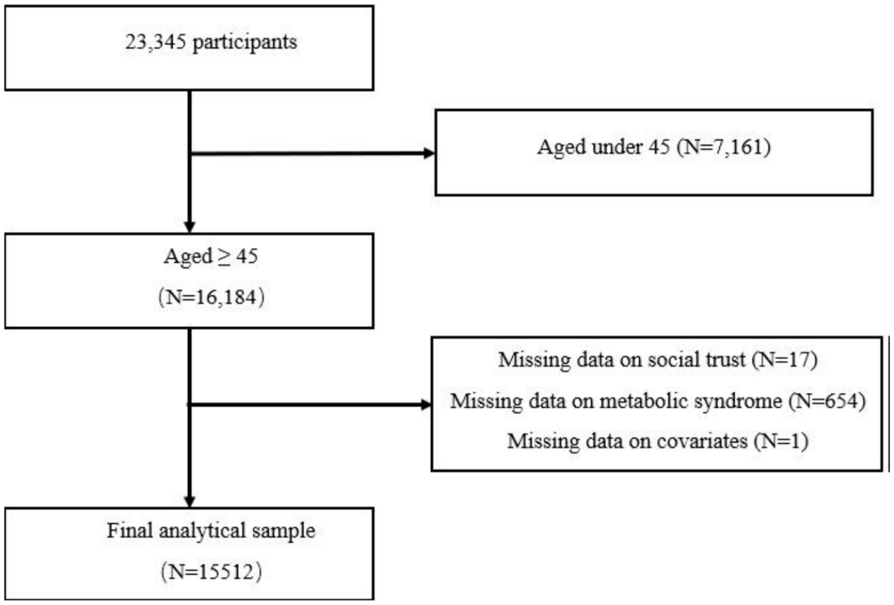

Figure S1. Analytical sample flowchart.

Table S1. Age-stratified association results.

	Age 45-59 years (N=9,554) OR (95% CI)		Age ≥60 years (N=5,958) OR (95% CI)	
	**Model 1**	**Model 2**	**Model 1**	**Model 2**
**Social trust**				
Low	Ref	Ref	Ref	Ref
Medium	0.74 (0.56-0.98)	0.73 (0.55-0.97)	0.90 (0.66-1.23)	0.89 (0.65-1.23)
High	0.73 (0.56-0.95)	0.74 (0.56-0.96)	0.90 (0.67-1.21)	0.89 (0.66-1.20)
Very high	0.89 (0.67-1.20)	0.90 (0.67-1.22)	0.76 (0.54-1.06)	0.74 (0.53-1.04)
**Smoking status**				
Never		Ref		Ref
Ever		1.41 (1.11-1.77)		1.39 (1.10-1.77)
Current		1.43 (1.22-1.67)		1.11 (0.89-1.38)
**Alcohol drinking**				
Never		Ref		Ref
Occasionally		0.86 (0.75-0.99)		0.89 (0.76-1.04)
<1/week		1.19 (0.90-1.57)		0.95 (0.62-1.45)
1-2/week		1.25 (0.97-1.60)		1.18 (0.80-1.74)
3-5/week		1.44 (1.13-1.84)		0.86 (0.58-1.28)
daily		0.89 (0.70-1.12)		0.84 (0.66-1.06)
**Physical activity**				
Never		Ref		Ref
1-3/month		1.00 (0.79-1.28)		1.32 (0.86-2.02)
1-2/week		1.06 (0.87-1.28)		0.95 (0.69-1.31)
3-5/week		1.01 (0.81-1.26)		1.35 (1.01-1.79)
Daily		1.06 (0.92-1.22)		1.21 (1.05-1.40)

Note. OR: odds ratio; CI: confidence interval; Ref: reference category; Model 1: Adjusted for age, gender, marital status, education, occupation, and household income, anxiety and depression; Model 2: Model 1 + health-related behaviors.

Table S2. Gender-stratified association results.

	Men (N=7,476) OR (95% CI)		Women (N=8,036) OR (95% CI)	
	**Model 1**	**Model 2**	**Model 1**	**Model 2**
**Social trust**				
Low	Ref	Ref	Ref	Ref
Medium	0.80 (0.60-1.07)	0.79 (0.59-1.06)	0.80 (0.59-1.10)	0.80 (0.59-1.10)
High	0.77 (0.59-1.01)	0.77 (0.59-1.01)	0.82 (0.61-1.10)	0.81 (0.61-1.09)
Very high	0.81 (0.60-1.09)	0.82 (0.60-1.11)	0.82 (0.59-1.14)	0.81 (0.58-1.12)
**Smoking status**				
Never		Ref		Ref
Ever		1.42 (1.20-1.69)		1.47 (0.40-5.39)
Current		1.35 (1.18-1.53)		1.33 (0.59-3.01)
**Alcohol drinking**				
Never		Ref		Ref
Occasionally		0.96 (0.83-1.11)		0.83 (0.71-0.97)
<1/week		1.29 (0.99-1.68)		0.59 (0.33-1.05)
1-2/week		1.32 (1.05-1.66)		0.71 (0.37-1.34)
3-5/week		1.30 (1.04-1.63)		1.13 (0.56-2.28)
daily		0.92 (0.76-1.11)		0.98 (0.59-1.62)
**Physical activity**				
Never		Ref		Ref
1-3/month		1.23 (0.95-1.59)		0.89 (0.60-1.30)
1-2/week		1.23 (1.00-1.52)		0.82 (0.62-1.10)
3-5/week		1.31 (1.05-1.64)		0.92 (0.69-1.22)
Daily		1.23 (1.07-1.41)		0.98 (0.84-1.13)

Note. OR: odds ratio; CI: confidence interval; Ref: reference category; Model 1: Adjusted for age, gender, marital status, education, occupation, household income, anxiety and depression; Model 2: Model 1 + health-related behaviors.
